# Floral nectar reabsorption and a sugar concentration gradient in two long-spurred *Habenaria* species (Orchidaceae)

**DOI:** 10.1186/s12870-023-04344-2

**Published:** 2023-06-22

**Authors:** Hai-Ping Zhang, Shi-Jia Wen, Hong Wang, Zong-Xin Ren

**Affiliations:** 1 Key Laboratory for Plant Diversity and Biogeography of East Asia, Kunming Institute of Botany, Chinese Academy of Sciences, Kunming, 650201 China; 2 University of Chinese Academy of Sciences, Beijing, China; 3 College of Forestry, Hainan University, Haikou, 570228 China; 4 Jiangxi Key Laboratory of Plant Resources and Biodiversity, Jingdezhen University, Jingdezhen, 334000 China; 5 Yunnan Lijiang Forest Biodiversity Observation and Research Station, Lijiang, 674100 China

**Keywords:** Nectar production, Nectar volume and concentration, Nectar reabsorption, Floral rewards, Flower age

## Abstract

**Background:**

Floral nectar is the most common reward flowers offered to pollinators. The quality and quantity of nectar produced by a plant species provide a key to understanding its interactions with pollinators and predicting rates of reproductive success. However, nectar secretion is a dynamic process with a production period accompanied or followed by reabsorption and reabsorption remains an understudied topic. In this study, we compared nectar volume and sugar concentration in the flowers of two long-spurred orchid species, *Habenaria limprichtii* and *H. davidii* (Orchidaceae). We also compared sugar concentration gradients within their spurs and rates of reabsorption of water and sugars.

**Results:**

Both species produced diluted nectar with sugar concentrations from 17 to 24%. Analyses of nectar production dynamics showed that as flowers of both species wilted almost all sugar was reabsorbed while the original water was retained in their spurs. We established a nectar sugar concentration gradient for both species, with differences in sugar concentrations at their spur’s terminus and at their spur’s entrance (sinus). Sugar concentration gradient levels were 1.1% in *H. limprichtii* and 2.8% in *H. davidii*, both decreasing as flowers aged.

**Conclusion:**

We provided evidence for the reabsorption of sugars but not water occurred in wilted flowers of both *Habenaria* species. Their sugar concentration gradients vanished as flowers aged suggesting a slow process of sugar diffusion from the nectary at the spur’s terminus where the nectar gland is located. The processes of nectar secretion/reabsorption in conjunction with the dilution and hydration of sugar rewards for moth pollinators warrant further study.

**Supplementary Information:**

The online version contains supplementary material available at 10.1186/s12870-023-04344-2.

## Background

Floral nectar production is central to reproductive success in most animal-pollinated angiosperms as nectar attracts possible pollen vectors with an easily digested resource [1, 2]. During the process of nectar consumption, animals are most likely to contact dehiscent anthers while depositing viable grains on receptive stigmas of a second compatible individual of the same species. Floral nectar is primarily a watery solution dominated by sugars with minor concentrations of additional nutrients including amino acids [3, 4], mineral ions, and vitamins [1, 2, 5–7]. Nectar sugars in most angiosperms are based primarily on varying ratios of sucrose and its monosaccharide [8].

However, as sugars provide both the primary energy resource and building blocks of cell walls for plants, their presence in floral nectar must represent a series of costs and trade-offs. Periods of active nectar secretion should be balanced by periods of reabsorption. If an individual plant increases sugar concentrations in their nectar, there may be less energy and resources available for the same plants to produce seeds [9]. Therefore, it is selectively advantageous for a plant to salvage and recycle the sugars and/or water in floral nectars that were not consumed by pollinators during floral life spans [10, 11].

Floral nectar reabsorption was first studied by Bonnier who described the process in flowers of *Platanthera* (Orchidaceae) [12]. It has since been shown to occur in other species from different families. Nepi and Stpiczyńska summarized the literature on nectar reabsorption and proposed three methods to record and document the process [10]. The first is observed and recorded as nectar volume and sugar concentrations decrease as the flowers age. The second requires a nectar substitution experiment, and the third uses radioactive tracers. We have updated their original literature database (Table S1), increasing the number of known species now known to reabsorb excess floral nectar. In addition, we include a significant number of species that fail to reabsorb their nectar [13]. By combining recent advances in microscopy with protocols for radioactive tracing of sugar transfer, our understanding of floral nectar production and regulation definitely improves [14–19]. However, we note that the most widely used, simplest, and easiest method to record nectar quality and quantity over time is to take the volumes and sugar concentrations of nectar secretions from flowers on the same plant over their respective life spans.

From plant-pollinator interaction and pollination aspects, it is also important to study floral nectar production, secretion dynamic and reabsorption. For most flowers, nectar is hidden in flowers, pollinators cannot detect the quantity and quality of floral nectar from a distance. After a pollinating animal visits a flower, the quality and quantity of floral nectar will determine if an animal continues to visit other flowers of the same plant or move to conspecific flowers of different individual plants or move to other plant species in the community. Therefore, it is suggested that a high variation of floral nectar attributes caused by secretion dynamic and reabsorption may be able to drive animals to move among flowers by partner manipulations [1, 7]. By this way, plants may get a high fitness by moving pollen grains from one plant to another plant through foraging by animals.

Furthermore, the natural secretion of sugars into nectar can occur passively along a concentration gradient influencing visiting behavior of insects. In 2007, Martins and Johnson provided evidence of gradients in nectar sugar concentration in five aerangoid orchid species [20]. These epidendroid orchids produce nectar in petal spurs at the bases of their labellum petals. As the nectar gland is located at the terminus of each spur the authors suspected that their sugar concentration gradients may occur passively due to the settlement of sugars within the solution inside the hollow spur [20]. A viscous drag in this narrow tube causes a slow mixing of sugar molecules with water in the nectar, or nectar reabsorption, if possible, at the upper part or entrance to the spur, known as the sinus. It has also been shown that nectar reabsorption is a specific function of the floral nectary in many plant species in angiosperms [10, 17–19]. Therefore, no nectar reabsorption can occur in species in which the nectary drips fluid and droplets are allowed to collect in a part of the flower disconnected from the nectary [13]. Nectar sugar gradients may function as a “sugar trail” encouraging pollinators to probe deeper into the flower further ensuring maximum contact between the pollinarium-carrying animal and the receptive lobes of the orchid’s stigma. As it backs out of the flower, following nectar consumption, the pollinator contacts the stigma’s rostellum triggering the release and dispersal of a second pollinarium. Regrettably, there are no similar published reports on sugar concentration gradients in other orchid plants with long floral spurs, and we do not know how floral nectar reabsorption may influence the dynamic of a sugar concentration gradient. We should expect that if nectar reabsorption occurs on or in the nectary as the flower ages, then any proposed sugar concentration gradient should decrease towards the end of a flower’s life span. Orchid flowers with spurs should make useful model systems to test this hypothesis.

*Habenaria* Willd. is the largest and most widespread terrestrial genus of orchids in the world with more than 800 accepted species [21]. The flowers in this genus produce spurs of varying lengths with nectar secreted at their bases. As nectar reabsorption has also been documented in the nectaries of related orchids (e.g., *Platanthera*) with aging spurs [16, 22–28], we expect that the nectary in the spurs of *Habenaria* species may also absorb sugar. *Habenaria* species are usually pollinated by members of the order Lepidoptera [29, 30]. *Habenaria limprichtii* Schltr. and *H. davidii* Franch. are two closely related species with long, nectar-secreting spurs. In recent years, there have been several detailed studies on their pollination and breeding systems [29–32]. As the flowering periods of these two species overlap, they provide an opportunity to test the hypothesis for nectar reabsorption and to establish and clarify sugar concentration gradients.

In this study, we investigated the variation of nectar production and sugar concentration during the floral life spans of both species. We address the following questions: (1) How do nectar production dynamics fluctuate during anthesis? (2) Does nectar reabsorption occur in either of the two species? (3) Is there a representative sugar concentration gradient at either end of the floral spur? (4) If a sugar gradient exists in either species, does it decrease as individual flowers age due to nectar reabsorption?

## Methods

### Study species and sites

**Fig. 1:**
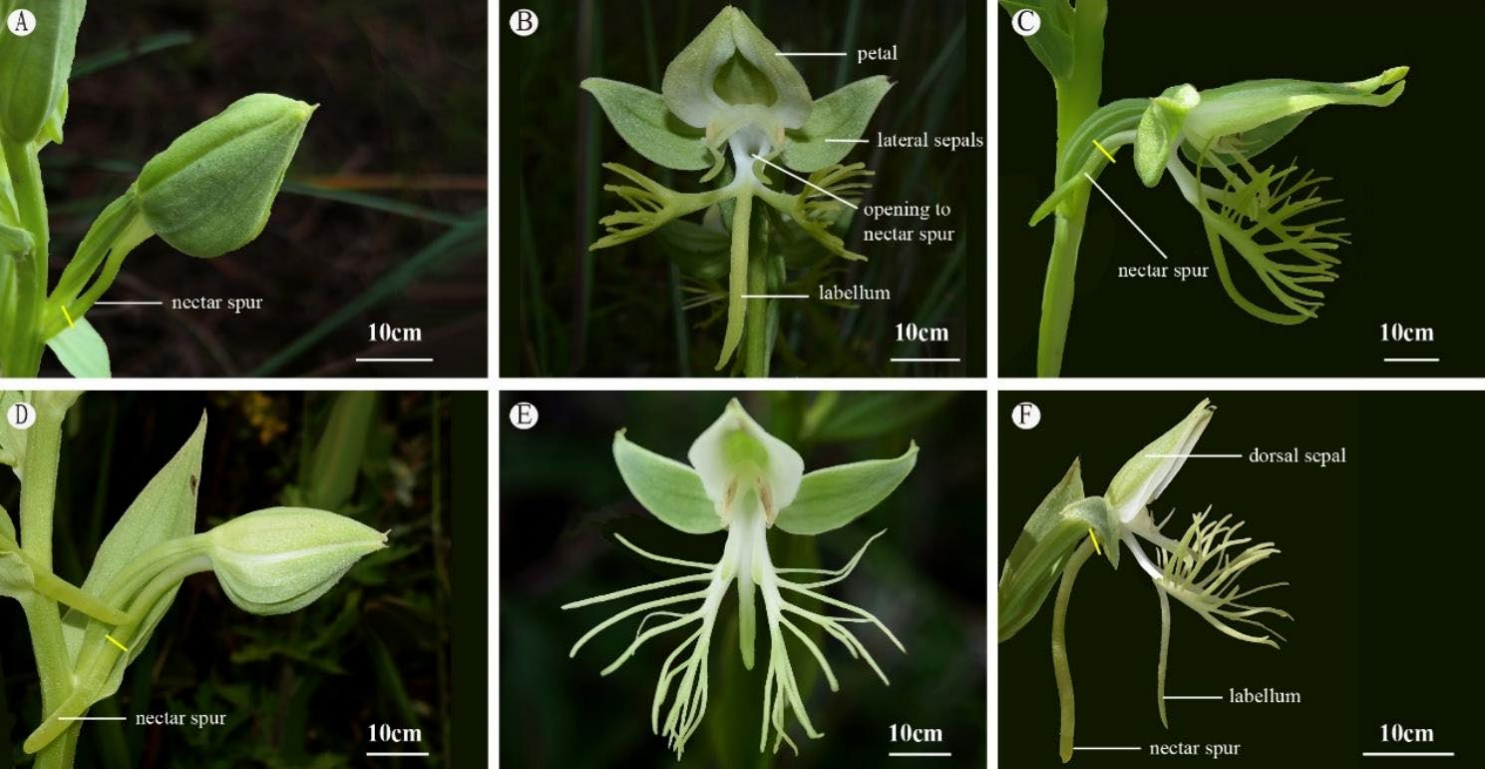
The floral morphology and nectar secreting spurs of two closely related *Habenaria* species in Yunnan, China. Floral nectar secretion begins during the bud stage and nectar volume is highest the first several days the perianth segments expand. The bud **A** and flower **B** of *H. limprichtii*; **C** the open flower of *H. limprichtii* with spur; The bud **D** and flower **E** of *H. davidii*; **F** the open flower of *H. davidii* showing its longer spur. Yellow lines indicate nectar levels inside the spurs

*Habenaria limprichtii* and *H. davidii* grow throughout southwestern China at elevations from 800 m a.s.l. up to 3500 m a.s.l. Both species have relatively large, white or greenish-white flowers (Fig. 1). Their nectar is produced inside their spurs and has little contact with the external environment. The length of the spur of *H. limprichtii* (20.33 ± 1.62 mm, N = 70; Fig. 1A, C;) is shorter than that of *H. davidii* (32.87 ± 3.31 mm, N = 79; Fig. 1D, F). Flower buds of each plant develop on a single flexible raceme and open almost simultaneously remaining receptive for 2–3 weeks. We did not find that a flower wilted quickly after its stigma received pollinia (Zhang, unpublished data) as in other orchid species [33]. Flowers of both species are visited by sphingids and other nocturnal moths [29–32] showing a “goodness of fit” between spur length and the length of proboscides of their sphingid pollinators [29]. This suggests that when a sphingid forages for flower nectar on these species, the tip of its proboscis reaches the terminus of the floral spurs ensuring that one of the two viscidia of the orchid’s rostellum will contact the moth’s eyes.

Field studies of nectar traits of these two *Habenaria* species were performed in 18 populations in Yunnan, China. Seven of them were localized in Kunming (MMJD, GM, MTL, TMQ, TS, ZN, LWS), two in Dali (BLS, TSC), six in Lijiang (G214, BSC, YSZ, YH, JQMK, ZT), and three in Shangri-La (SG, LG, ALC). For additional details on experimental field sites, see Table S2. The experiments of nectar reabsorption was conducted in the MTL and TS populations of *H. limprichtii*, and MMJD and TMQ populations of *H. davidii*. Field experiments were conducted from August - September 2020 and 2021. Nectar sugar concentration gradients were collected from August to October 2021 from 18 wild populations of both *Habenaria* species.

Both species were not listed as endangered or protected, and these study sites are not in nature reserves. All flowers were collected without damaging the individual plant. Fieldwork conducted in Yunnan and specimen collections were permitted by the Germplasm Bank of Wild Species, Kunming Institute of Botany, Chinese Academy of Sciences based on the national guidelines and legislation following the rules of the Convention on the Trade in Endangered Species of Wild Fauna and Flora (https://www.cites.org/). Voucher specimens of both species were identified by Dr. Zong-Xin Ren and deposited in the herbarium of the Kunming Institute of Botany (KUN), Chinese Academy of Sciences, Kunming, China.

### Nectar sampling

During the flowering season of both studied species, we randomly selected opened flowers (one flower per inflorescence) encountered in the population during the afternoon (17:00 to 18:30) of each day when the temperature was between 18–25℃. Then we removed selected flowers from their plants, and placed them into a Styrofoam container with wet wipes on the bottom, then we brought them back to the field lab. We measured floral nectar at night from 19:30 to 23:00 in the field lab under artificial light. One flower per individual raceme was used to compare the nectar traits of *H. limprichtii* (N = 75 plants, in 2020; N = 281 plants, in 2021) and *H. davidii* (N = 70 plants, in 2020; N = 299 plants, in 2021). The nectar column height of each spur was measured with a digital caliper (error: 0.01 mm), and the nectar was withdrawn using graduated glass microcapillary tubes (1 µl, 5 µl, 10 µl, 20 µl minicaps, accuracy: 0.5%, coefficient of variation: 1.0%; Hirschmann Laboratory, Germany) following the method of Tao et al. [29]. Nectar volume was calculated from the nectar column height in microcapillary tubes. Sugar concentration was obtained with a manual refractometer (0 to 50% brix, Bellingham & Stanley Ltd., UK). To calculate sugar mass per unit volume (mg/µl), we used the equation “Y = 0.00226 + 0.00937X + 0.0000585 × ^2”^ (see [34]), where X is the sugar concentration (%). The total sugar (mg) per flower was determined by calculating nectar volume (µl) times sugar mass per unit volume(mg/µl).

To determine the pattern of nectar production over a flower’s lifespan, we recorded floral nectar attributes (volume and sugar concentration) using multiple flowers of both species in 2020 and 2021 that were previously isolated from moths under nylon bags for the duration of the floral life span. In addition, to determine the age of the flower before we picked it and took its nectar, we individually marked the date each flower bud opened using a plastic label. We then also recorded the age of each flower we measured based on the number of days following the opening of the perianth segments. We divided the flowering period into three stages. The first stage lasts for 1–10 days. During this stage, the amount of nectar secretion will gradually increase until it reaches its peak around the tenth day of flowering. This is followed by the second phase, 10–15 days after the flowers open. During this stage, nectar secretion begins to gradually decrease. The third stage, 15–20 days after the flowers open, the petals start to turn into black. In 2020, we measured floral nectar dynamics by monitoring 30 plants (180 flowers) of *H. limprichtii* from 26th August to 16th September, and 15 plants (85 flowers) of *H. davidii* from 23rd August to 16th September in intervals from one to six days. In 2021, we repeated the same sampling by increasing sample sizes taking measurements over 48 h (two days) intervals. Nectar was collected from a total of 36 racemes representing 218 flowers of *H. limprichtii* and 29 racemes representing 209 flowers of *H. davidii*. When measuring nectar traits, we randomly selected flowers from the bottom to the top of the inflorescence.

To test if the nectar sugar concentration gradient was self-consistent throughout the length of the spurs of each species, we cut each spur into sections (1-2 mm) then extracted the nectar out of each section using graduated microcapillary tubes (1 µl, 5 µl, 10 µl or 20 µl, Hirschmann Laboratory, Germany). Volumes and sugar concentration from each section were recorded and correlated to the section of spur length and its distance from the opening (sinus) of the spur. To accurately characterize differences in sugar concentration gradients between our two species, we calculated the difference in sugar concentration between the bottom (terminus) and top (sinus) of the nectar column within each spur.

### Data analyses

#### Comparative nectar production in two co-blooming *Habenaria* species

Nectar variation among populations of the same species were not considered in this study when conducting the comparative analysis of nectar traits between species. Instead, we pooled data collected for each species from each of their populations each year. Differences among species and years in nectar traits were tested using one-way ANOVA followed by Tukey’s post-hoc test. This was analyzed with R software and then plotted with the *ggplot2* package [35].

#### Nectar reabsorption

Due to the different sampling intervals for nectar reabsorption in 2020 and 2021, we performed statistical analyses separately for each year. To determine the dynamics of nectar secretion across the lifespan of each flower species, we employed the *ggplot2* package in R to generate boxplots [35]. The relationship between nectar traits (nectar volume, sugar concentration, and total sugar per flower) and flower age in 2020 and 2021 was determined using linear regression.

#### Analysis of nectar concentration gradients

The relationship between nectar sugar concentration and the distance from the terminus to the sinus of a spur was calculated and analyzed. We estimated the gradient of sugar concentration along the length of the nectar column at different ages of the two *Habenaria* species by constructing a slope chart using the R-package *ggplot2* [35]. To examine the bicontinuous interaction relationship between two variables, we fitted a multiple linear regression model with interactions using the “stargazer” and “effects” functions available in the R package *car* [36]. This approach allowed us to capture the joint effects of the variables, while also accounting for any possible interaction effects. Data related to differences in sugar concentration between the bottom and top of the nectar column inside the spur were pooled together for detailed analysis, and a mean was given with standard deviation (SD).

All analyses were performed with the R statistical environment (R 4.1.2) [37].

## Results

### Comparison of nectar traits between two species

We observed nectar accumulation in spurs of both species before their perianth segments expanded to expose the column (Fig. 1A and D), with the total length of the nectar column height within the spur varying according to species. The nectar column of *H. limprichtii* rose only from 0.33 to 0.66 of the length of the spur (Fig. 1C), while nectar levels in *H. davidii* tended to fill up almost to the entire length of the spur the day the bud fully opened (Fig. 1F). The mean nectar volume of 11 populations of *H. limprichtii* varied between 0.07 and 25.05 µl, and the average sugar concentration in the nectar was 24.4 ± 6.01%. For *H. davidii*, each flower in 12 populations produced between 0.07 and 35.28 µl of nectar, with a sugar concentration of 17.75 ± 7.36%. These values corresponded to ca. 1.41 ± 1.26 mg of total sugar per flower of *H. limprichtii*, and 2.88 ± 2.59 mg of total sugar per flower of *H. davidii* (Table 1).

**Table 1:** Interspecific variation between nectar column height, nectar volume, sugar concentration and the total sugar (sugar mass or weight) per flower of two *Habenaria* species in 2020 and 2021. Nectar trait data, *H. limprichtii* (data from TS and MTL populations in 2020, N = 75, and 11 populations in 2021, N = 281), *H. davidii* (data from MMJD and TMQ populations in 2020, N = 70; and 12 populations in 2021, N = 299)

Nectar traits	Year	*H. limprichtii*	*H. davidii*	*df*	*F*	*P*
Nectar column height (mm)	2020	7.71 ± 4.69	24.63 ± 12.36	1	121.9	0.001
	2021	5.61 ± 3.83	11.41 ± 8.84	1	102.7	0.001
Nectar volume (µl)	2020	8.11 ± 7.25	26.01 ± 15.51	1	84.49	0.001
	2021	5.41 ± 4.79	15.75 ± 12.30	1	173.7	0.001
Sugar concentration (%)	2020	19.15 ± 9.37	14.84 ± 7.14	1	9.629	0.01
	2021	24.42 ± 6.01	17.75 ± 7.36	1	141.8	0.001
Total sugar per flower (mg/flower)	2020	1.69 ± 1.92	4.09 ± 3.24	1	29.79	0.001
	2021	1.41 ± 1.26	2.88 ± 2.59	1	73.79	0.001

All nectar traits differed significantly between these two species. The measurement results for two consecutive years showed that there were significant differences in nectar column height (F = 121.9, d.f. = 1, P < 0.001 in 2020, Fig. 2A; F = 102.7, d.f. = 1, P < 0.001 in 2021, Fig. 2E), nectar volume (F = 84.49, d.f. = 1, P < 0.001 in 2020, Fig. 2B; F = 102.7, d.f. = 1, P < 0.001 in 2021, Fig. 2F), sugar concentration (F = 9.629, d.f. = 1, P < 0.01 in 2020, Fig. 2C; F = 141.8, d.f. = 1, P < 0.001 in 2021, Fig. 2G) and total sugar concentration per flower (F = 29.79, d.f. = 1, P < 0.001, in 2020, Fig. 2D; F = 73.79, d.f. = 1, P < 0.001 in 2021, Fig. 2H) between *H. limprichtii* and *H. davidii*.

**Fig. 2:**
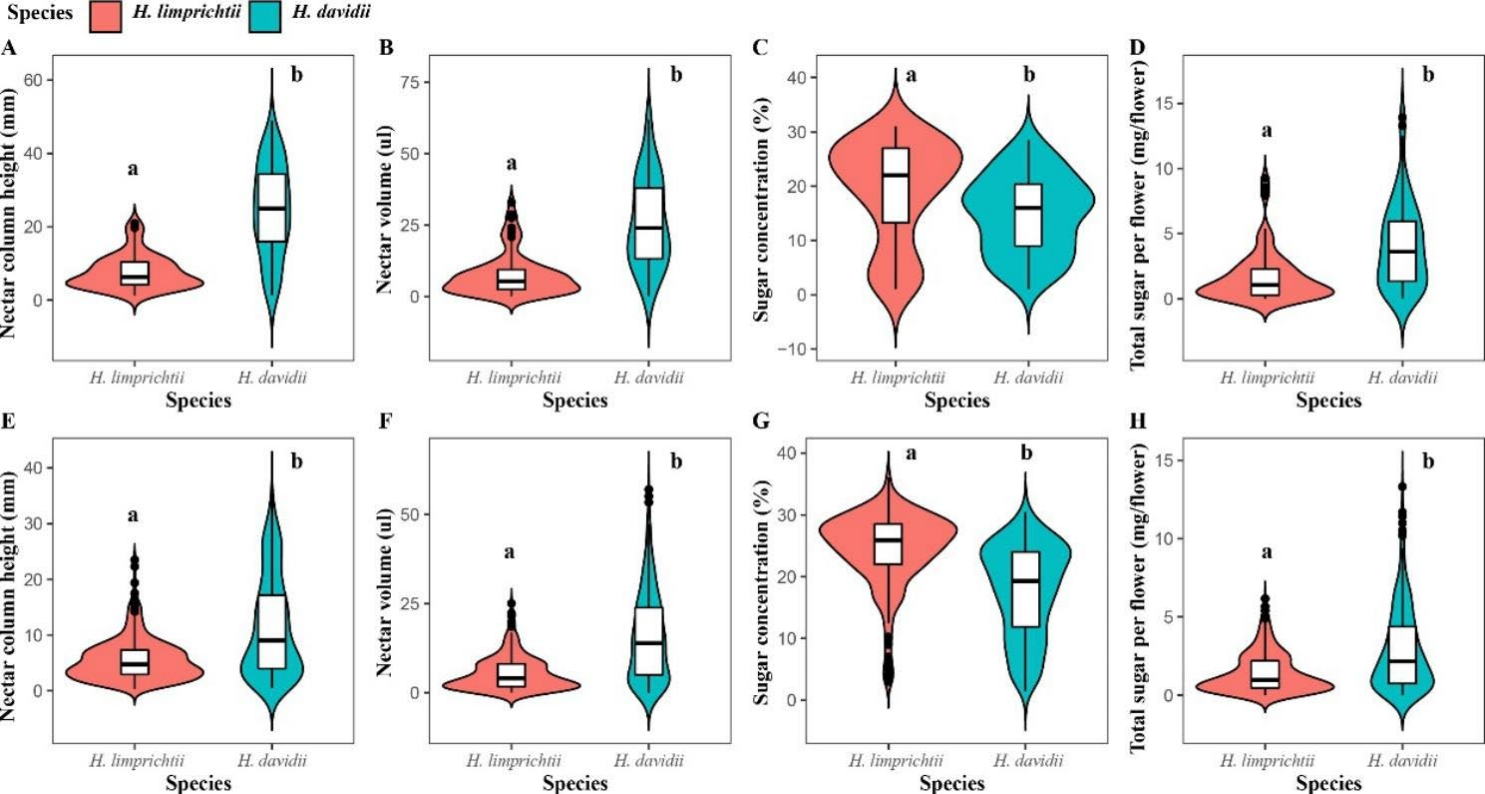
Comparison of nectar traits of two *Habenaria* species. **A** Comparison of nectar column height of two *Habenaria* species in 2020; **B** Comparison of nectar volume of two *Habenaria* species in 2020; **C** Comparison of sugar concentration in nectar of two *Habenaria* species in 2020; **D** Comparison of dissolved sugar per flower of two *Habenaria* species in 2020; **E** Comparison of nectar column height in spurs of two *Habenaria* species in 2021; **F** Comparison of nectar volume of two *Habenaria* species in 2021; **G** Comparison of sugar concentration of nectar from two *Habenaria* species in 2021; **H** Comparison of the total sugar per flower of two *Habenaria* species in 2021. Different lower case letter indicates significant difference (P < 0.01)

### Nectar reabsorption

**Fig. 3:**
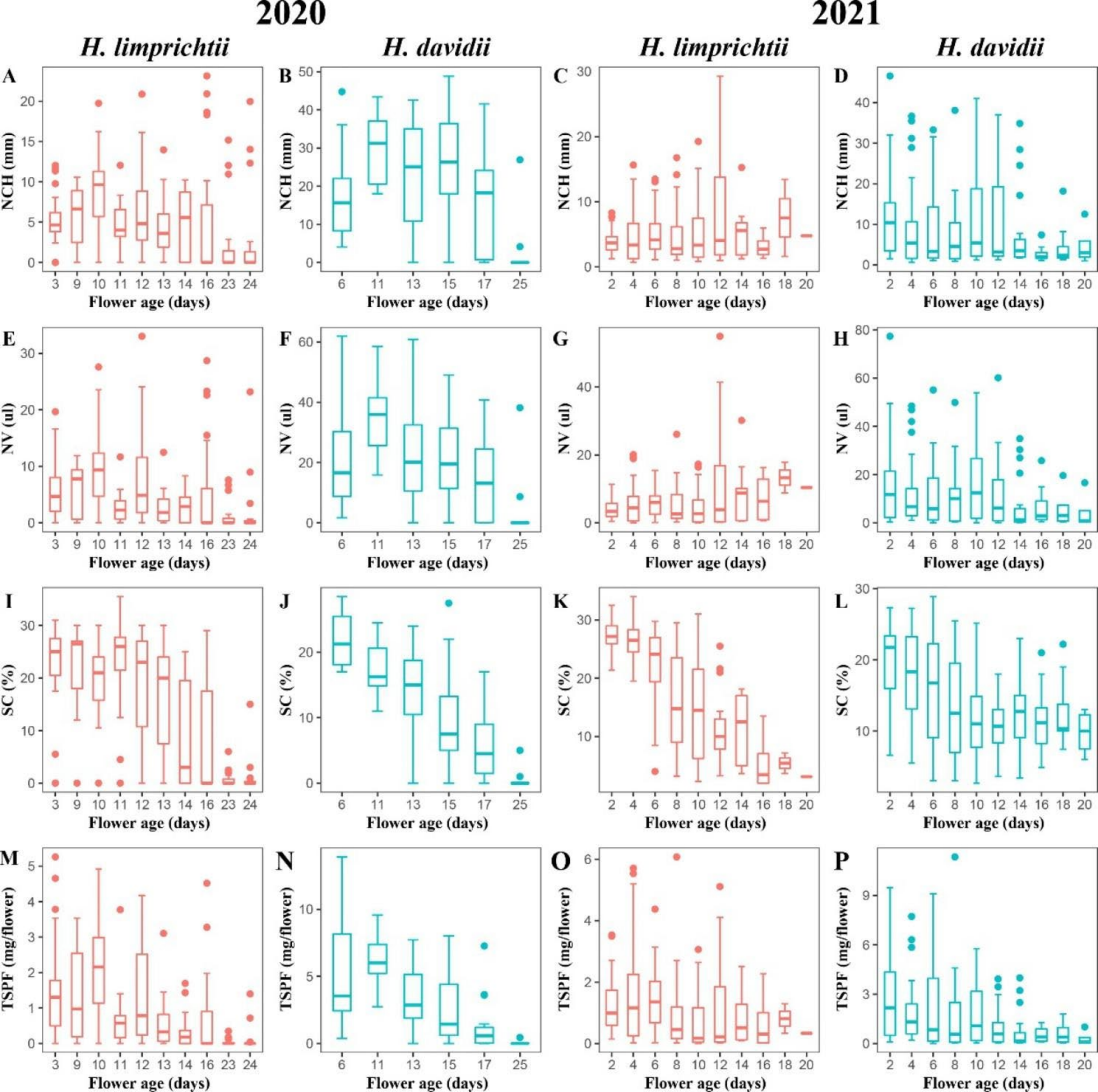
Temporal pattern of nectar secretion during anthesis in two *Habenaria* species. **A-D** Variation in the nectar column height of two *Habenaria* species in 2020 and 2021; Variation in the nectar column height of **(A)***H. limprichtii* and **(B)***H. davidii* in 2020; Variation in the nectar column height of **(C)***H. limprichtii* and **(D)***H. davidii* in 2021; **E-H** Variation in the nectar volume of two *Habenaria* species in 2020 and 2021; Variation in the nectar volume of **(E)***H. limprichtii* and **(F)***H. davidii* in 2020; Variation in the nectar volume of **(G)***H. limprichtii* and **(H)***H. davidii* in 2021; **I-L** Variation in the sugar concentration of two *Habenaria* species in 2020 and 2021; Variation in the sugar concentration of **(I)***H. limprichtii* and **(J)***H. davidii* in 2020; Variation in the sugar concentration of **(K)***H. limprichtii* and **(L)***H. davidii* in 2021; **M-P** Variation in the total sugar per flower of two *Habenaria* species in 2020 and 2021; Variation in the total sugar per flower of **(M)***H. limprichtii* and **(N)***H. davidii* in 2020; Variation in the total sugar per flower of **(O)***H. limprichtii* and **(P)***H. davidii* in 2021. NCH: Nectar column height; NV: Nectar volume; SC: Sugar concentration; TSPF: Total sugar per flower

Nectar secretion dynamics in the two *Habenaria* species are illustrated in Fig. 3. Both species start nectar production before anthesis so that the sugar in the nectar does not accumulate slowly, but directly drops from the highest level (Fig. 3; Fig. S1). An asynchronization between petal opening and nectar secretion has been shown in these two studied species, which results in floral visitors that can easily consume a ready amount of nectar at the beginning of the anthesis.

The mean nectar column height within the spurs of both *Habenaria* species was not significant and fluctuated in 2020 and 2021 (Fig. 3A, B, C, D). In flowers of both *Habenaria* species, nectar volume was affected slightly by flower age (Fig. 3E, F, G, H). However, this decline was so minor that the accumulated nectar volume throughout the flowering period remained broadly constant (Fig. S1A, B, C, D). Such as the *H. limprichtii* in 2020, the average of nectar volume decreases from 5.48 µl to 2.42 µl, this magnitude of change (3.06 µl) is small. The total nectar volume within all the flowers on the same raceme began to decline after about 10 days for *H. davidii* and 12 days for *H. limprichtii*.

Flowers of both *Habenaria* species showed variation in sugar concentration with floral age because the mean nectar sugar concentration decreased significantly as flowers aged (Fig. S1E, F, G, H). Based on correlation coefficients, sugar concentration was negatively correlated with flower age, especially for *H. davidii* in 2020 (*R*^*2*^ = 0.61, *P* < 0.001) and *H. limprichtii* in 2021 (*R*^*2*^ = 0.5127, *P* < 0.001). Differences in sugar concentration in both species were most significant between the early flowering stage and the late flowering stage in 2020 and 2021 (Fig. 3I, J, K, L). For example, the concentration of nectar in *H. limprichtii* decreased from 27.32% in the initial flowering to 3.1% in the late flowering stage.

The total amount of sugar per flower also varied over the floral lifespan in both species. Sugar production was greatest during the first 10 days, then declined gradually (Fig. 3M, N, O, P). In general, the samples obtained at the end of the anthesis tended to contain lower concentrations of sugars compared to newly opened flowers. The total sugar per flower has a linear decreasing tendency (*P* < 0.01) with increasing flower age (Fig. S1I, J, K, L). The largest decreases in the total sugar per flower were observed at *H. davidii* in 2020, and ranged from 5.482 mg/flower to 0.088 mg/flower.

### Nectar sugar concentration gradients

**Fig. 4:**
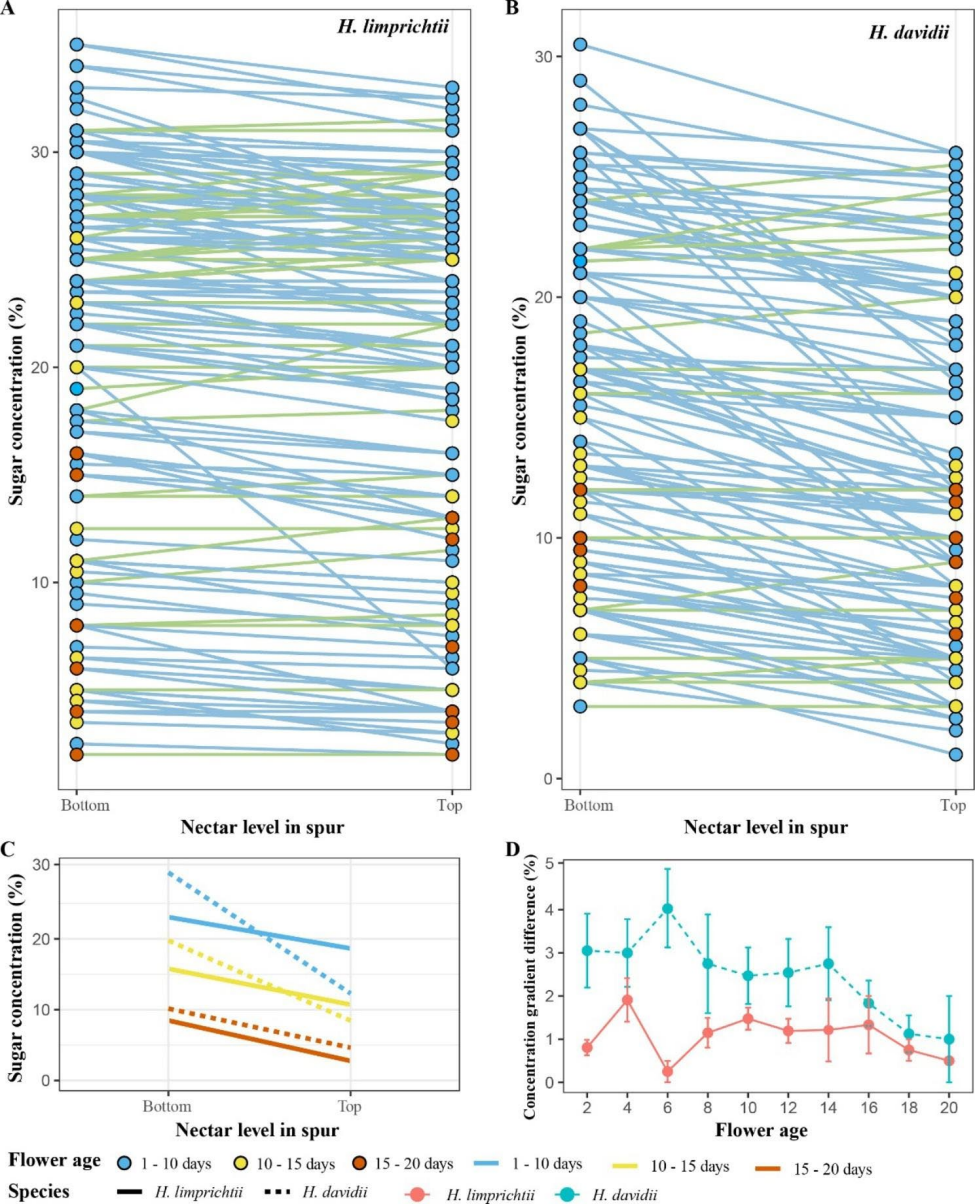
Nectar sugar concentration gradients in the spurs of two *Habenaria* species. **A-B** Comparison of sugar concentration between the bottom and top of the nectar column at different floral ages in *H. limprichtii* and *H. davidii*. Images show three floral ages; 1–10 days (blue circle), 10–15 days (yellow circle) and 15–20 days (orange circle), respectively. Samples with higher sugar concentrations at the bottom of nectar column (spur terminus) than that at the top (spur sinus) are shown with blue lines, otherwise, with green lines. **C** Comparison of variation of sugar concentrations from the bottom to the top of the nectar column in spurs at different floral ages of two *Habenaria* species. The image shows three floral ages, 1–10 days (blue line), 10–15 days (yellow line) and 15–20 days (orange line), respectively. Species are identified by different line shapes, with the solid line representing *H. limprichtii* and the dashed line representing *H. davidii*. **D** Comparison of the variation in concentration gradient differences with floral age between two *Habenaria* species

Gradients in nectar sugar concentrations along the length of the nectar column in spurs were observed in both *Habenaria* species (Fig. 4). The height of the nectar column in the spur varied between species (Fig. 1; Table 1). Mostly, the nectar at the bottom (spur base/terminus) of most nectar columns had higher sugar concentrations than the nectar at the top (sinus) of the column in both species (Fig. 4). There were some exceptions in some nectar columns taken from *H. limprichtii* in which there were no discernible differences in sugar concentrations at the top versus the bottom of the spur (Fig. 4A, B).

For both species, the sugar concentration at each stage of flowering significantly decreased with nectar level in the spur and as floral age increased (Fig. 4C). During the first stages (about1-10 days) of the floral life span, sugar concentration decreased significantly with nectar column height in the spur from bottom to top. In contrast, sugar concentration did not show such a sharp decline after 10 days (Fig. 4C). Specifically, we found that nectar sugar concentration decreased slightly during mid-flowering (10–15 days) and again in the oldest surviving flowers (15–20 days).

As flowers of *H. limprichtii* aged, the differences in concentration gradients showed a progressive increase, peaking on the 4th day of the floral life span (Fig. 4D). This difference in gradients decreased after day 4, reaching its minimum on day 6, then decreased again but slowly after a slight increase (1.1% on average). In contrast, the difference in concentration gradients in *H. davidii* plants was at their maximum during the first 6 days of the floral life span and then they showed a gradual decline (2.8% on average; Fig. 4D). Compared to *H. limprichtii*, the sugar concentration difference between the bottom and top of the nectar column in *H. davidii* are considerably more prominent (Fig. S2).

## Discussion

### Do floral nectar volumes and concentrations vary between two co-blooming *Habenaria* species?

Previous publications suggest that nectars of hawkmoth pollinated flowers have sugar concentration ranging from 14.1 to 31.9% [38]. The concentrations recorded for our two *Habenaria* species fell within this range. It suggests that sugar production in spurs of both species is held at levels typical of a standard suite of adaptive traits associated with a classical, nocturnal, sphingid-pollination syndrome, which seems convergent with other orchid species and unrelated angiosperms with the same putative pollination group [29, 30, 39]. The cost of foraging during cool nights must be high for flying, poikilothermic moths that regulate their body temperatures due to the heat produced by the friction they produce by rapidly flapping of their wings [40–42]. It is still unclear whether the chemical energy gained from the nectar of our orchid species is sufficient to support the moth’s foraging activities. Further investigation is needed to determine whether more concentrated sources of sugar are available in the communities, which could potentially shed new light on the foraging behavior of these moths.

Our results also showed significant differences in nectar production between the two *Habenaria* species, with *H. davidii* producing more than twice the amount of sugar than *H. limprichtii*. Such differences are possibly related to differences in floral size. In general, large flowers produce greater amounts of sugars [43]. While both species shared one species of hawkmoth pollinator (*Deilephila elpenor* subsp. *lewisii*), previous floral choice experiments suggested that this hawkmoth showed a high degree of floral constancy that interspecific visitations between the two orchid species were uncommon [30]. Therefore, it is safe to suspect that floral nectar difference between two species along other floral traits, floral scent may contribute to floral choice and a high fidelity of hawkmoth species to each of both orchid species. Tao et al. [10] used the rate of embryo development in the seeds as an indicator of cross- and self- pollination, they found that a high proportion of fruits from a population of *H. limprichtii* was resulted from self pollination. Multiple populations with different nectar attribute variation within individual plants and among individual plants should be used to examine out-crossing rates for the fruits, to answer if floral nectar variation does drive moths to move between individual plants.

### Do aging flowers of two *Habenaria* species reabsorb nectar sugar and water?

In our study, nectar reabsorption starts around day 10 during anthesis, as indicated by the decrease in nectar volume and total sugar per flower (Fig. 3). While both *Habenaria* species reabsorbed only a little nectar water, their senescent flowers recycled most of the remaining sugars. This indicates that while both nectar water and sugars were reabsorbed together at the same time, sugar reabsorption might be preferential. However, we cannot exclude the possibility that any slight drop in nectar volume was due to evaporation.

How does one explain the imbalance of water versus sugar reabsorption? In general, nectar production, including its secretion and reabsorption, requires a great expenditure in plant energy incurring costs [44–46]. Therefore, it is more worthwhile if the investment cost is low for an individual plant. Maximizing the reabsorption of all unconsumed sugar is an effective way to reduce metabolic costs. In the case of our *Habenaria* species the sugar reclaimed by the aging flower could be reinvested in the production of seeds or stored in fleshy subterranean organs during the upcoming period of dormancy. Recycling water is of lesser importance as both species bloom during the monsoon season and are usually associated with perpetually moist substrates [30].

One of the distinct flowering features of both studied species is that all the buds in a plant open almost simultaneously within one or two days, remaining receptive for 2–3 weeks. Buds about to open were full of nectar in the spurs of both species. Such nectar volume can last more than 10 days without a decrease in volume when these flowers are isolated to prevent moth visits. As the spurs of both species are too narrow to insert pipettes for removal experiments, we don’t know whether either species replenishes nectar in spurs over the floral lifespan. Based on our current data, we did not find a clear trend that flowers continue to secrete but we did record a trend towards slightly lower nectar volumes, possibly due to evaporation of water or reabsorption. Tao et al. [29] measured the diurnal and nocturnal nectar production of *H. limprichtii* but did not find any differences between samples. This is consistent with our current findings that following initial secretions as flowers open, their spurs do not continue to secrete either sugar or water.

Previous studies found that nectar reabsorption can be induced by pollination and/or floral wilting. The rate of sugar reabsorption from nectar increases after pollination, allowing the recycling of nutrients to the development of ovules and fruit walls [10, 11, 47, 48]. Koopowitz and Marchant observed that the nectar from an unpollinated African epiphytic orchid (*Aerangis verdickii*) was not reabsorbed [49]. Contrary to their results, we found that nectar reabsorption was unrelated to pollination in both *Habenaria* species, as nectar reabsorption in our two species also occurs in the absence of pollination. Other previous studies also did not observe obvious floral senescence after hand pollination for both species [29, 30]. We suspect that the stigma surfaces of species in the genus *Habenaria* may encourage multiple visits by pollinators leading to multiple self- (including geitonogamous) and cross-pollinations as pollen-masses from different flowers, belonging to the same multi-flowering plant and different phenotypes, accumulate repeatedly on such long-lived lobes. Indeed, Tao et al. [29] did find a high inbreeding depression in one of our populations from Lijiang with outcrossed pollinated fruits containing a high proportion of well-developed embryos.

Though nectar reabsorption has been frequently reported (60 species; see Table S1), species representing only 30 families are currently documented, and information on nectar traits and the extent of nectar reabsorption remains scarce for most plant groups. To fill in knowledge about the mechanisms of nectar reabsorption at the cell and molecular level, for example, breakdown and transport pathways of floral nectar under reabsorption [11, 28, 50], it is necessary to clarify the dynamics of nectar production and the transport of sugars in more species. We have demonstrated that the *H. limprichtii* and *H. davidii* reabsorbed all or most of the nectar, but it is unclear where the nectar is absorbed and how transactional these processes actually are.

### Are there nectar sugar concentration gradients in spurs of both *Habenaria* species?

In this study, we found a clear nectar sugar concentration gradient for both species. The average level of the nectar sugar concentration gradient was 1.1% in *H. limprichtii* and 2.8% in *H. davidii*. Sugar concentration is greater at the bottom of the spur for two reasons. First, the nectar gland that releases sugar molecules is located at the spur’s terminus. Second, as sugar molecules are heavier than water molecules they will tend to gravitate towards the bottom due to the slow process of diffusion inside a narrow cylinder and the absence of stirring the liquid (no cilia lining in this spur). Furthermore, we suspect that the viscosity of nectar should also cause a lower sugar molecule dispersal within the water. The viscosity of sugar varies among different sugar compositions, and is positively related to sugar concentration, while negatively related to temperature [51]. The highest viscosity of sugar is sucrose at a low temperature, so we expected a high viscosity for the nectar of our two *Habenaria* species, because at night the environmental temperature is around 10–15℃ at most of our study sites. The viscosity of nectar is high for *H. davidii* as it has a high nectar concentration. Corbet et al. [52] also proposed that the viscous surface layer of viscous nectar solutions may help retard further evaporation. In our case, if either nectariferous *Habenaria* plants were observed to have a noticeably viscous nectar surface layer, its “skin” will contribute to slowing the rate of equilibration between air humidity and the sugar solution. Given these points, it should be safe to expect that the slow mixing of nectar and water due to viscous drag in the narrow spur is one of the reasons for the nectar concentration gradient. These gradients are maintained by diffusion-limited adhesion. Furthermore, if the viscosity of different sugars constrains their mixing with water, we should expect that the nectar in the upper part of the nectar column (nearest to the sinus) will have a higher ratio of hexose (glucose + fructose) to sucrose. At the bottom (terminus) of the spur, the ratio will be reversed, and the nectar will be sucrose dominant.

A nectar sugar gradient means that the same flower is offering two kinds of rewards to two prospective pollinators. The moth with the shorter proboscis receives the most dilute or weakest reward, but the orchid receives the less efficient, “short-tongued” pollinator. Tao et al. [29] did find that short-tongued noctuids had to push their bodies into the flowers of *H. limprichtii*, but less likely to remove pollinarium indicated by moth scales and haris left on the viscidium disk. In contrast, the moth with the longest proboscis has access to a thicker or more viscous syrup. The sugar concentration gradient in the long spur may drive the moth to extend its proboscis to the terminus of the spur. Indeed, moths can detect the concentration gradients in the nectar spur since they are capable of perceiving sugar concentration changes as low as 0.1% or lower [53]. As Darwin showed in the case of *Angraecum sesquipedale*, the moth that pushes and struggles to probe the terminus of the spur is most likely to remove and/or deposit the pollinaria. In our species, Tao et al. [29] found a perfect match between the distance between the viscidia to the terminus of the spur and the length of proboscides of hawkmoths suggesting the proboscides of hawkmoths did reach the bases of spurs ensuring the attachment of pollinaria attaching to the insect’s eyes.

However, such a nectar gradient is not solely related to the length of floral spurs, but may also be related to other factors, such as the twisted morphology of the spurs [20, 54], and the biochemical/ cytological/molecular mechanisms of nectar secretion and sugar reabsorption [10, 28, 55]. It is also safe to predict that sugar concentration may be common in many families of angiosperms with flowers that have spurs or a single, narrow, elongated, and tubular perianth. It would be most interesting to compare sugar concentrations in European species of bee-pollinated *Aquilegia* (with their short spurs) *versus* the American species with long spurs pollinated by hummingbirds or sphingid moths depending on location and color.

### Does floral nectar reabsorption cause sugar concentration gradient to disappear in aged flowers?

Martins and Johnson suspected that the concentration gradient should be associated with nectar reabsorption in four orchid species [20]. They suggested that a sugar concentration gradient may be also due to sugar secretion from the nectary (at the terminus of the spur) and/ or reabsorption from the upper part of the spur. We find this explanation unlikely as the nectar gland is the only place known so far where nectar is reabsorbed in any flower. We found greater nectar concentration gradients in the earlier stages of flower anthesis (1–10 days), which may be due simply to the act of secretion overriding reabsorption at this stage in the floral lifespan while our own sugar gradient decreased slowly as our flowers. At this time, in contrast, nectar sugar reabsorption was strong (see above). This result confirmed our hypothesis that the sugar concentration gradient along a spur will decrease as the nectar at the spur’s terminus of spur begins to reabsorb nectar.

## Conclusions

We explored the patterns of variation in nectar production related to flower age in two closely related orchid species. Our analyses showed that nectar secretion may occur concomitantly with nectar reabsorption continuing after secretion has ended. When considering nectar production dynamics, analyses showed that both *Habenaria* species reabsorbed almost all nectar sugars in the spur. We also detected differences in sugar concentration gradients between our two species. The concentration gradients in *H. davidii* were greater than in *H. limprichtii*, as the longer spur of *H. davidii* may force visitors to probe more deeply into spurs improving the plant’s reproductive success. Our results indicate that nectar reabsorption may play a role in shaping concentration gradients. It is still not known, however, where and how nectar is reabsorbed by the gland, how molecular mechanisms transport recycled sugars to other plant tissues and what is the functional significance of these concentration gradients.

## Electronic supplementary material

Below is the link to the electronic supplementary material.

Supplementary Material 1

## Data Availability

All data generated or analyzed during this study are included in this published article.
