# Supplementary material for: Floral nectar reabsorption and a sugar concentration gradient in two long-spurred Habenaria species (Orchidaceae)
Source: BMC Plant Biol. 2023 Jun 22;23:331. doi: 10.1186/s12870-023-04344-2 (PMC10286501; doi:10.1186/s12870-023-04344-2)
Supplement: Supplementary file 1 — Supplementary Material 1 [file 12870_2023_4344_MOESM1_ESM.docx]

# Supplemental files

**Table S1** Examples of nectar resorption in angiosperms updating Table 1 in Nepi and Stpiczyńska (2008a) with additional species in blue. cp: Cumulative sugar production in repeatedly sampled protected flowers; sc: Sugar production in standing crop of protected flowers. NA：Not assessed.

| **Family** | **Species** | **Method(s)** | **Reabsorption** | | **Reference** |
| --- | --- | --- | --- | --- | --- |
|  |  |  | **Water** | **Sugar** |  |
| Amaryllidaceae | *Allium cepa* | Sugar decrease | - | + | Kumar & Kumar Gupta, 1993 |
| Apiaceae | *Carum carvi* | Volume and sugar decrease | + | + | Langenberger & Davis, 2002 |
| Apiaceae | *Daucus carota* | Radioactive tracer | NA | + | Ziegler & Lüttge, 1959 |
| Apocynaceae | *Mandevilla pentladiana* | Volume and sugar decrease | + | + | Torres & Galetto, 1998 |
| Asphodelaceae | *Aloe castanea* | Volume and sugar decrease | + | + | Nicolson & Nepi, 2005 |
| Asteraceae | *Echinacea pallida var. angustifolia* | Volume and sugar decrease | + | + | Wist & Davis, 2008 |
|  | *Echinacea purpurea* | Volume and sugar decrease | + | + | Wist & Davis, 2008 |
| Bignoniaceae | *Pyrostegia venusta* | Nectar substitution | NA | NA | Galetto et al., 1994 |
| Boraginaceae | *Borago officinalis* | Nectar removal | - | - | Búrquez & Corbe,t 1991 |
|  | *Cerinthe major* | cp vs sc; Sugar decrease | - | + | Gilbert et al., 1991; Nocentini et al., 2012 |
|  | *Buglossoides purpurocaerulea* | Volume and sugar decrease | + | + | Nocentini et al., 2013 |
|  | *Echium plantagineum* | Sugar decrease | - | + | Corbet & Delfosse, 1984 |
| Brassicaceae | *Brassica napus* | cp vs sc; Nectar removal | - | + | Búrquez & Corbet, 1991 |
| Bromeliaceae | *Billbergia distachia* | Nectar removal; Electron microscope observation of nectaries | - | + | Zambon et al., 2020 |
|  | *Tillandsia deppeana* | Nectar removal | - | - | Ordano & Ornelas, 2004 |
|  | *Tillandsia multicaulis* | Nectar removal | - | - | Ordano & Ornelas, 2004 |
|  | *Pseudalcantarea viridiflora* | Volume and sugar decrease | + | + | Aguilar-Rodríguez et al., 2022 |
| Campanulaceae | *Campanula isophylla* | Radioactive tracer | NA | + | Kartashova & Tsytlenok, 1968 |
| Caryophyllaceae | *Silene*sp. | Volume decrease | + | - | Witt et al., 1999 |
|  | *Silene dioica* (male flowers) | Volume and sugar decrease | + | + | Hemborg, 1998 |
| Combretaceae | *Combretum fruticosum* | Volume and sugar decrease | + | + | Bernardello et al. 1994 |
| Cucurbitaceae | *Cucurbita maxima* | Volume and sugar decrease | + | + | Ashworth & Galetto, 2002 |
|  | *Cucurbita pepo* | Volume and sugar decrease; Radioactive tracer; Nectar substitution; Electron microscope observation of nectaries | + | + | Nepi et al., 1996a; Nepi et al., 1996b, 2001, 2011; Nepi & Stpiczyńska, 2007; Cardoso-Gustavson et al., 2013; Nepi & Stpiczyńska, 2008a |
| Ericaceae | *Rhododendron decorum* | Volume and sugar decrease | + | + | Pyke et al., 2020 |
| Euphorbiaceae | *Euphorbia tithymaloides* | Volume and sugar decrease | + | + | Veiga Blanco et al., 2013 |
| Fabaceae | *Mucuna japira* | Volume and sugar decrease | + | + | Agostini et al., 2011 |
|  | *Mucuna urens* | Volume and sugar decrease | + | + | Agostini et al., 2011 |
|  | *Inga sessilis* | Volume and sugar decrease | + | + | Amorim et al., 2013 |
|  | *Sophora fernandeziana* | Volume and sugar decrease | + | + | Bernardello et al., 2004 |
|  | *Vicia faba* | Radioactive tracer | NA | + | Cardoso-Gustavson & Davis, 2015 |
|  | *Trifolium repens* | Volume and sugar decrease | + | + | Jakobsen & Kristjansson, 1994 |
|  | *Baptisia australis* | Radioactive tracer | NA | + | Kartashova & Tsytlenok, 1968 |
|  | *Phaseolus multiflorus* | Radioactive tracer | NA | + | Kartashova & Tsytlenok, 1968 |
|  | *Hymenaea stigonocarpa* | Electron microscope observation of nectaries | - | + | Paiva et al., 2008 |
|  | *Anagrys foetida* | Volume and sugar decrease | + | + | Valtueña et al., 2007 |
| Gentianaceae | *Swertia bimaculata* | Volume and sugar decrease | + | + | Wang et al., 2018 |
| Geraniaceae | *Geranium sylvaticum* | Sugar decrease | - | + | Varga et al., 2013 |
| Grossulariaceae | *Ribes dikuscha* | Radioactive tracer | NA | + | Kartashova & Tsytlenok, 1968 |
|  | *Ribes nigrum* | Radioactive tracer | NA | + | Kartashova & Tsytlenok, 1968 |
| Liliaceae | *Fritillaria imperialis* | Nectar removal | - | - | Búrquez & Corbet, 1991 |
|  | *Fritillaria meleagris* | Volume and sugar decrease，Electron microscope observation of nectaries | + | + | Stpiczyńska et al., 2012 |
| Loranthaceae | *Ligaria cuneifolia* | Sugar decrease | - | + | Galetto et al., 1990; Riveira et al., 1996 |
|  | *Psittacanthus auriculatus* | Volume and sugar decrease | + | + | Pérez‐Crespo et al., 2016 |
| Malvaceae | *Hibiscus sinensis* | Radioactive tracer | NA | + | Cardoso-Gustavson & Davis, 2015 |
|  | *Gossypium hirsutum* | Radioactive tracer | NA | + | Cardoso-Gustavson & Davis, 2015 |
| Myrtaceae | *Eucalyptus*sp*.* | Volume and sugar decrease | + | + | Davis, 1997 |
|  | *Leptospermum polygalifolium* | Sugar decrease | - | + | Obeng-Darko et al., 2022 |
| Orchidaceae | *Anathallis obovata* | Radioactive tracer | NA | + | Arévalo-Rodrigues et al., 2021 |
|  | *Bonatea polypodantha* | Volume and sugar decrease | + | + | Balducci et al., 2020 |
|  | *Aerangis verdickii* | Volume and sugar decrease | + | + | Koopowitz & Marchant, 1998 |
|  | *Mystacidium venosum* | Volume and sugar decrease | + | + | Luyt & Johnson, 2002 |
|  | *Octomeria crassifolia* | Radioactive tracer | NA | + | Arévalo-Rodrigues et al., 2021 |
|  | *Platanthera chlorantha* | Radioactive tracer; Electron microscope observation of nectaries | NA | + | Stpiczyńska, 2003ab, Nep i& Stpiczyńska, 2007; Stpiczyńska et al., 2005 |
| Plantaginaceae | *Linaria vulgaris* | Volume and sugar decrease | + | + | Nepi et al., 2003 |
| Proteaceae | *Grevillea robusta* | cp vs sc; nectar substitution | + | + | Nicolson, 1995 |
| Rosaceae | *Cerasus cerasoides* | Volume and sugar decrease | + | + | Dong et al., 2016 |
| Solanaceae | *Datura ferox* | Volume and sugar decrease | + | + | Torres et al., 2013 |
|  | *Jaltomata quipuscoae* | Nectar removal | - | - | Mione et al., 2020 |
| Valerianaceae | *Centranthus ruber* | Radioactive tracer | NA | + | Cardoso-Gustavson & Davis, 2015 |
| Vochysiaceae | *Qualea grandiflora* | Measurement of nectar production dynamics | - | - | Potascheff et al., 2020 |

Method applied for the reference search.

In September of 2022, we conducted an additional Web of Science search for nectar reabsorption that included the following words and terms: topic: “floral nectar” and “reabsorption” or topic: “nectar production dynamics” and “reabsorption”/ “resorption”. The search returned 43 results and 10 results, respectively. We excluded all repetitive, irrelevant literature, and added some relevant articles that were not retrieved by Nepi and Stpiczyńska (2008a) before 2007 in addition to new publications that appeared after 2008.

References for Table S1.

Agostini K, Sazima M, Galetto L. Nectar production dynamics and sugar composition in two *Mucuna* species (Leguminosae, Faboideae) with different specialized pollinators. Naturwissenschaften. 2011; 98(11):933-942. <https://doi.org/10.1007/s00114-011-0844-6>.

Aguilar-Rodríguez PA, Tschapka M, García-Franco JG, Krömer T, López-Acosta JC, MacSwiney GMC. Intraspecific variability of nectar attracts different bats: the case of *Pseudalcantarea viridiflora*, a bromeliad with crepuscular anthesis. Acta Bot Bras. 2022; 35:597-611. <https://doi.org/10.1590/0102-33062020abb0503>.

Amorim FW, Galetto L, Sazima M. Beyond the pollination syndrome: nectar ecology and the role of diurnal and nocturnal pollinators in the reproductive success of *Inga sessilis* (Fabaceae). Plant Biol. 2013; 15(2):317-327. <https://doi.org/10.1111/j.1438-8677.2012.00643.x>.

Arévalo-Rodrigues G, de Barros F, Davis AR, Cardoso-Gustavson P. Floral glands in myophilous and sapromyophilous species of Pleurothallidinae (Epidendroideae, Orchidaceae)—osmophores, nectaries, and a unique sticky gland. Protoplasma. 2021; 258(5):1061-1076. <https://doi.org/10.1007/s00709-021-01624-2>.

Ashworth L, Galetto L. Differential nectar production between male and female flowers in a wild cucurbit: *Cucurbita maxima* ssp. *andreana* (Cucurbitaceae). Can J Bot. 2002; 80(11):1203-1208. <https://doi.org/10.1139/b02-110>.

Balducci MG, Van der Niet T, Johnson SD. Diel scent and nectar rhythms of an African orchid in relation to bimodal activity patterns of hawkmoth pollinators. Ann Bot. 2020; 126(7):1155-1164. <https://doi.org/10.1093/aob/mcaa132>.

Bernardello G, Aguilar R, Anderson GJ. The reproductive biology of *Sophora fernandeziana* (Leguminosae), a vulnerable endemic species from Isla Robinson Crusoe. Am J Bot. 2004; 91(2):198-206. <https://doi.org/10.3732/ajb.91.2.198>.

Bernardello L, Galetto L, Rodriguez IG. Reproductive biology, variability of nectar features and pollination of *Combretum fruticosum* (Combretaceae) in Argentina. Bot J Linn Soc. 1994; 114(3):293-308. <https://doi.org/10.1111/j.1095-8339.1994.tb01938.x>.

Búrquez A, Corbet SA. Do flowers reabsorb nectar?. Funct Ecol. 1991; 369-379. <https://doi.org/10.2307/2389808>.

Cardoso‐Gustavson P, Davis AR. Is nectar reabsorption restricted by the stalk cells of floral and extrafloral nectary trichomes?. Plant Biol. 2015; 17(1):134-146. <https://doi.org/10.1111/plb.12208>.

Cardoso-Gustavson P, Aguiar JMRBVA, Ricardo Pansarin E, de Barros F. A light in the shadow: the use of Lucifer Yellow technique to demonstrate nectar reabsorption. Plant Methods. 2013; 9(1):1-7. <https://doi.org/10.1186/1746-4811-9-20>.

Corbet SA, Delfosse ES. Honeybees and the nectar of *Echium plantagineum* L. in southeastern Australia.  Aust J Ecol. 1984; 9(2):125-139. https://doi.org/10.1111/j.1442-9993.1984.tb01351.x.

Davis AR. Influence of floral visitation on nectar-sugar composition and nectary surface changes in Eucalyptus. Apidologie. 1997; 28(1):27-42. <https://doi.org/10.1051/apido:19970104>.

Dong K, Dong Y, Su R, Zhang JL, Qing Z, Yang XC, Ren XX, Ma YB, He SY. Effect of nectar reabsorption on plant nectar investment in *Cerasus cerasoides*. Curr Sci. 2016; 251-256. <https://www.jstor.org/stable/24906753>.

Galetto L, Bernardello LM, Juliani HR. Acerca del nectario, nectary visitantes florales en *Ligaria cuneifolia* (Loranthaceae). Darwiniana. (1990); 155-161. <https://www.jstor.org/stable/23222525>.

Galetto L, Bernardello LM, Juliani HR. Characteristics of secretion of nectar in *Pyrostegia venusta* (Ker‐Gawl.) Miers (Bignoniaceae). New Phytol. 1994; 127(3):465-471. <https://doi.org/10.1111/j.1469-8137.1994.tb03964.x>.

Gilbert FS, Haines N, Dickson K. Empty flowers. Funct Ecol. 1991; 29-39. <https://doi.org/10.2307/2389553>.

Hemborg ÅM. Seasonal dynamics in reproduction of first-year females and males in *Silene dioica*. Int J Plant Sci. 1998; 159(6):958-967. <https://doi.org/10.1086/314095>.

Jakobsen HB, Kristjansson K. Influence of temperature and floret age on nectar secretion in *Trifolium repens* L. Ann Bot. 1994; 74:327–334. <https://doi.org/10.1006/anbo.1994.1125>.

Koopowitz H, Marchant TA. Postpollination nectar reabsorption in the African epiphyte *Aerangis verdickii* (Orchidaceae). Am J Bot. 1998; 85(4):508-512. <https://doi.org/10.2307/2446434>.

Kratashova N, Tsylenok S. On the biologic role of nectaries and nectar. II. Studies on the resorption of nectar by flower parts, SIB OTD Akao Nauk. SSSR Ser. Biol. Med. Nauk. 1968; 5:134-137.

Kumar J, Gupta JK. Nectar sugar production and honeybee foraging activity in 3 species of onion (Allium species). Apidologie. 1993; 24(4):391-396. <https://doi.org/10.1051/apido:19930405>.

Langenberger MW, Davis AR. Temporal changes in floral nectar production, reabsorption, and composition associated with dichogamy in annual caraway (*Carum carvi*; Apiaceae). Am J Bot. 2002; 89(10):1588-1598. <https://doi.org/10.3732/ajb.89.10.1588>.

Luyt R, Johnson SD. Postpollination nectar reabsorption and its implications for fruit quality in an epiphytic orchid. Biotropica. 2002; 34(3):442-446. <https://doi.org/10.1111/j.1744-7429.2002.tb00558.x>.

Mione T, Diaz IA. Dracula's mistress: removal of blood-red floral nectar results in secretion of more nectar. Plant Ecol Evol. 2020; 153(1):59-66. <https://doi.org/10.5091/plecevo.2020.1589>.

Nepi M, Ciampolini F, Pacini E. Development and ultrastructure of *Cucurbita pepo* nectaries of male flowers. Ann Bot. 1996a; 78(1):95-104. <https://doi.org/10.1006/anbo.1996.0100>.

Nepi M, Cresti L, Guarnieri M, Pacini E. Dynamics of nectar production and nectar homeostasis in male flowers of *Cucurbita pepo* L. Int J Plant Sci. 2011; 172(2):183-190. <https://doi.org/10.1086/657648>.

Nepi M, Guarnieri M, Pacini E. Nectar secretion, reabsorption, and sugar composition in male and female flowers of *Cucurbita pepo*. Int J Plant Sci. 2001; 162(2):353-358. <https://doi.org/10.1086/319581>.

Nepi M, Pacini E, Nencini C, Collavoli E, Franchi GG. Variability of nectar production and composition in *Linaria vulgaris* (L.) Mill.(Scrophulariaceae). Plant Syst Evol. 2003; 238(1):109-118. <https://doi.org/10.1007/s00606-002-0275-0>.

Nepi M, Pacini E, Willemse MTM. Nectary biology of *Cucurbita pepo*: ecophysiological aspects. Acta Bot Neerl. 1996b; 45(1):41-54.

Nepi M, Stpiczyńska M. Nectar resorption and translocation in *Cucurbita pepo* L. and *Platanthera chlorantha* Custer (Rchb.). Plant Biol. 2007; 9(01):93-100. <https://doi.org/10.1055/s-2006-924287>.

Nepi M, Stpiczyńska M. The complexity of nectar: secretion and resorption dynamically regulate nectar features. Naturwissenschaften. 2008a; 95:177–184. <https://doi.org/10.1007/s00114-007-0307-2>.

Nicolson SW. Direct demonstration of nectar resorption in the flowers of *Grevillea robusta* (Proteaceae). Funct Ecol. 1995; 9:584–588. https://doi.org/10.2307/2390148.

Nicolson SW, Nepi M. Dilute nectar in dry atmospheres: nectar secretion patterns in *Aloe castanea* (Asphodelaceae). Int J Plant Sci. 2005; 166(2):227-233. <https://doi.org/10.1086/427616>.

Nocentini D, Pacini E, Guarnieri M, Martelli D, Nepi M. Intrapopulation heterogeneity in floral nectar attributes and foraging insects of an ecotonal Mediterranean species. Plant Ecol. 2013; 214(6):799-809. <https://doi.org/10.1007/s11258-013-0204-z>.

Nocentini D, Pacini E, Guarnieri M, Nepi M. Flower morphology, nectar traits and pollinators of *Cerinthe major* (Boraginaceae-Lithospermeae). Flora-Morphology, Distribution, Functional Ecology of Plants. 2012; 207(3):186-196. <https://doi.org/10.1016/j.flora.2012.01.004>.

Obeng-Darko SA, Brooks PR, Veneklaas EJ, Finnegan PM. Sugar and dihydroxyacetone ratios in floral nectar suggest continuous exudation and reabsorption in *Leptospermum polygalifolium* Salisb. Plant Sci. 2022; 323:111378. <https://doi.org/10.1016/j.plantsci.2022.111378>.

Ordano M, Ornelas JF. Generous-like flowers: nectar production in two epiphytic bromeliads and a meta-analysis of removal effects. Oecologia. 2004; 140(3):495-505. https://doi.org/10.1007/s00442-004-1597-0.

Paiva EAS, Machado SR. The floral nectary of *Hymenaea stigonocarpa* (Fabaceae, Caesalpinioideae): structural aspects during floral development. Ann Bot. 2008; 101(1):125-133. <https://doi.org/10.1093/aob/mcm268>.

Pérez‐Crespo MJ, Ornelas JF, Martén‐Rodríguez S, González‐Rodríguez A, Lara C. Reproductive biology and nectar production of the Mexican endemic *Psittacanthus auriculatus* (L oranthaceae), a hummingbird‐pollinated mistletoe. Plant Biol. 2016; 18(1):73-83. https://doi.org/10.1111/plb.12365.

Potascheff CdM, de Brito VL, Galetto L, Sebbenn AM, Oliveira PE. Nectar features, diurnal and nocturnal pollinators, and male fitness in *Qualea grandiflora* (Vochysiaceae). Plant Syst Evol. 2020; 306(1):1-12. <https://doi.org/10.1007/s00606-020-01646-y>.

Pyke GH, Ren ZX, Trunschke J, Lunau K, Wang H. Salvage of floral resources through re-absorption before flower abscission. Sci Rep. 2020; 10(1):1-9. <https://doi.org/10.1038/s41598-020-72994-5>.

Rivera GL, Galetto L, Bernardello L. Nectar secretion pattern, removal effects, and breeding system of *Ligaria cuneifolia* (Loranthaceae). Can J Bot. 1996; 74(12):1996-2001. <https://doi.org/10.1139/b96-239>.

Stpiczyńska M. Nectar resorption in the spur of *Platanthera chlorantha* Custer (Rchb.) Orchidaceae–structural and microautoradiographic study. Plant Syst Evol. 2003a; 238(1):119-126. <https://doi.org/10.1007/s00606-002-0281-2>.

Stpiczyńska M. Incorporation of [^3^H] sucrose after the resorption of nectar from the spur of *Platanthera chlorantha* (Custer) Rchb. Can J Bot. 2003b; 81(9):927-932. <https://doi.org/10.1139/b03-085>.

Stpiczyńska M, Milanesi C, Faleri C, Cresti M. Ultrastructure of the nectary spur of *Platanthera chlorantha* (Custer) Rchb.(Orchidaceae) during successive stages of nectar secretion. Acta Biol Cracov Ser Bot. 2005; 47(2):111-119.

Stpiczyńska M, Nepi M, Zych M. Secretion and composition of nectar and the structure of perigonal nectaries in *Fritillaria meleagris* L.(Liliaceae). Plant Syst Evol. 2012; 298(5):997-1013. <https://doi.org/10.1007/s00606-012-0609-5>.

Torres C, Galetto L. Patterns and implications of floral nectar secretion, chemical composition, removal effects and standing crop in *Mandevilla pentlandiana* (Apocynaceae). Bot J Linn Soc. 1998; 127(3):207-223. <https://doi.org/10.1111/j.1095-8339.1998.tb02098.x>.

Torres C, Mimosa M, Galetto L. Nectar ecology of *Datura ferox* (Solanaceae): an invasive weed with nocturnal flowers in agro-ecosystems from central Argentina. Plant Syst Evol. 2013; 299(8): 1433-1441. <https://doi.org/10.1007/s00606-013-0805-y>.

Valtuena FJ, Ortega-Olivencia A, Rodriguez-Riano T. Nectar production in *Anagyris foetida* (Fabaceae): two types of concentration in flowers with hanging droplet. Int J Plant Sci. 2007; 168(5):627-638. <https://doi.org/10.1086/513482>.

Varga S, Nuortila C, Kytöviita MM. Nectar sugar production across floral phases in the *gynodioecious protandrous* plant *Geranium sylvaticum*. PLoS One. 2013; 8(4):e62575. <https://doi.org/10.1371/journal.pone.0062575>.

Veiga Blanco T, Galetto L, Machado IC. Nectar regulation in *Euphorbia tithymaloides* L., a hummingbird‐pollinated Euphorbiaceae. Plant Biol. 2013; 15(5):910-918. <https://doi.org/10.1111/j.1438-8677.2012.00695.x>.

Wang S, Fu WL, Du W, Zhang Q, Li Y, Lyu YS, Wang XF. Nectary tracks as pollinator manipulators: The pollination ecology of *Swertia bimaculata* (Gentianaceae). Ecol Evol. 2018; 8(6):3187-3207. <https://doi.org/10.1002/ece3.3838>.

Wist TJ, Davis AR. Floral nectar production and nectary anatomy and ultrastructure of *Echinacea purpurea* (Asteraceae). Ann Bot. 2006; 97(2):177-193. <https://doi.org/10.1093/aob/mcj027>.

Wist TJ, Davis AR. Floral structure and dynamics of nectar production in *Echinacea pallida* var. *angustifolia* (Asteraceae). Int J Plant Sci. 2008; 169(6):708-722. https://doi.org/10.1086/533602.

Witt T, Jürgens A, Geyer R, Gottsberger G. Nectar dynamics and sugar composition in flowers of *Silene* and *Saponaria* species (Caryophyllaceae). Plant Biol. 1999; 1(03):334-345. <https://doi.org/10.1055/s-2007-978524>.

Zambon V, Agostini K, Nepi M, Rossi ML, Martinelli AP, Sazima M. The role of nectar traits and nectary morphoanatomy in the plant-pollinator interaction between *Billbergia distachia* (Bromeliaceae) and the hermit *Phaethornis eurynome* (Trochilidae). Bot J Linn Soc. 2020; 192(4):816-827. <https://doi.org/10.1093/botlinnean/boz107>.

Ziegler H, Lüttge U. Über die Resorption von C^14^-Glutaminsäure durch sezernierende Nektarien. Naturwissenschaften. 1959; 46(5):176-177. <https://doi.org/10.1007/BF00629741>.

**Table S2** Geographical locations of 18 sites containing populations of one or both *Habenaria* species. Field studies of measured nectar traits were conducted in 4 populations (*H. limprichtii*: MTL, TS; *H. davidii*: MMJD, TMQ) in 2020 and 18 populations (*H. limprichtii*: GM, MTL, TMQ, TS, ZN, LWS, BLS, TSC, YSZ, YH; *H. davidii*: MMJD, TMQ; *H. davidii*: MMJD, GM, MTL, TMQ, LWS, G214, BSC, JQMK, ZT, SG, LG, ALC) in 2021.

| **Location** | **Species** | **Geographical locations** | **Altitude (m)** |
| --- | --- | --- | --- |
| MMJD, Kunming | *H. davidii* | 25°12′38″, 102°44′42″ | 2276 |
| GM, Kunming | *H. limprichtii* | 25°10′45″, 102°43′54″ | 2260 |
|  | *H. davidii* | 25°10′53″, 102°43′57″ | 2297 |
| MTL, Kunming | *H. limprichtii* | 25°12′23″, 102°45′12″ | 2501 |
|  | *H. davidii* | 25°12′35″, 102°44′33″ | 2313 |
| TMQ, Kunming | *H. limprichtii* | 25°12′37″, 102°46′22″ | 2282 |
|  | *H. davidii* | 25°11′44″, 102°47′26″ | 2166 |
| TS, Kunming | *H. limprichtii* | 25°15′51″, 102°43′28″ | 2418 |
| ZN, Kunming | *H. limprichtii* | 25°21′58″, 102°48′40″ | 2138 |
| LWS, Kunming | *H. limprichtii* | 24°44′54″, 102°53′34″ | 2668 |
|  | *H. davidii* | 24°45′15″, 102°52′17″ | 2375 |
| BLS, Dali | *H. limprichtii* | 26°09′34″, 99°54′25″ | 2253 |
| TSC, Dali | *H. limprichtii* | 26°36′38″, 99°50′54″ | 2574 |
| G214, Lijiang | *H. davidii* | 26°37′50″, 99°57′30″ | 2319 |
| BSC, Lijiang | *H. davidii* | 26°51′18″, 100°11′04″ | 2438 |
| YSZ, Lijiang | *H. limprichtii* | 26°59′32″, 100°11′43″ | 2730 |
| YH, Lijiang | *H. limprichtii* | 27°01′42″, 100°12′57″ | 2848 |
| JQMK, Lijiang | *H. davidii* | 27°02′08″, 100°15′20″ | 2797 |
| ZT, Lijiang | *H. davidii* | 26°58′04″, 100°14′08″ | 2533 |
| SG, Shangri-La | *H. davidii* | 26°53′06″, 100°00′16″ | 1847 |
| LG, Shangri-La | *H. davidii* | 26°59′40″, 99°59′21″ | 2189 |
| ALC, Shangri-La | *H. davidii* | 27°12′01″, 99°52′21″ | 2261 |


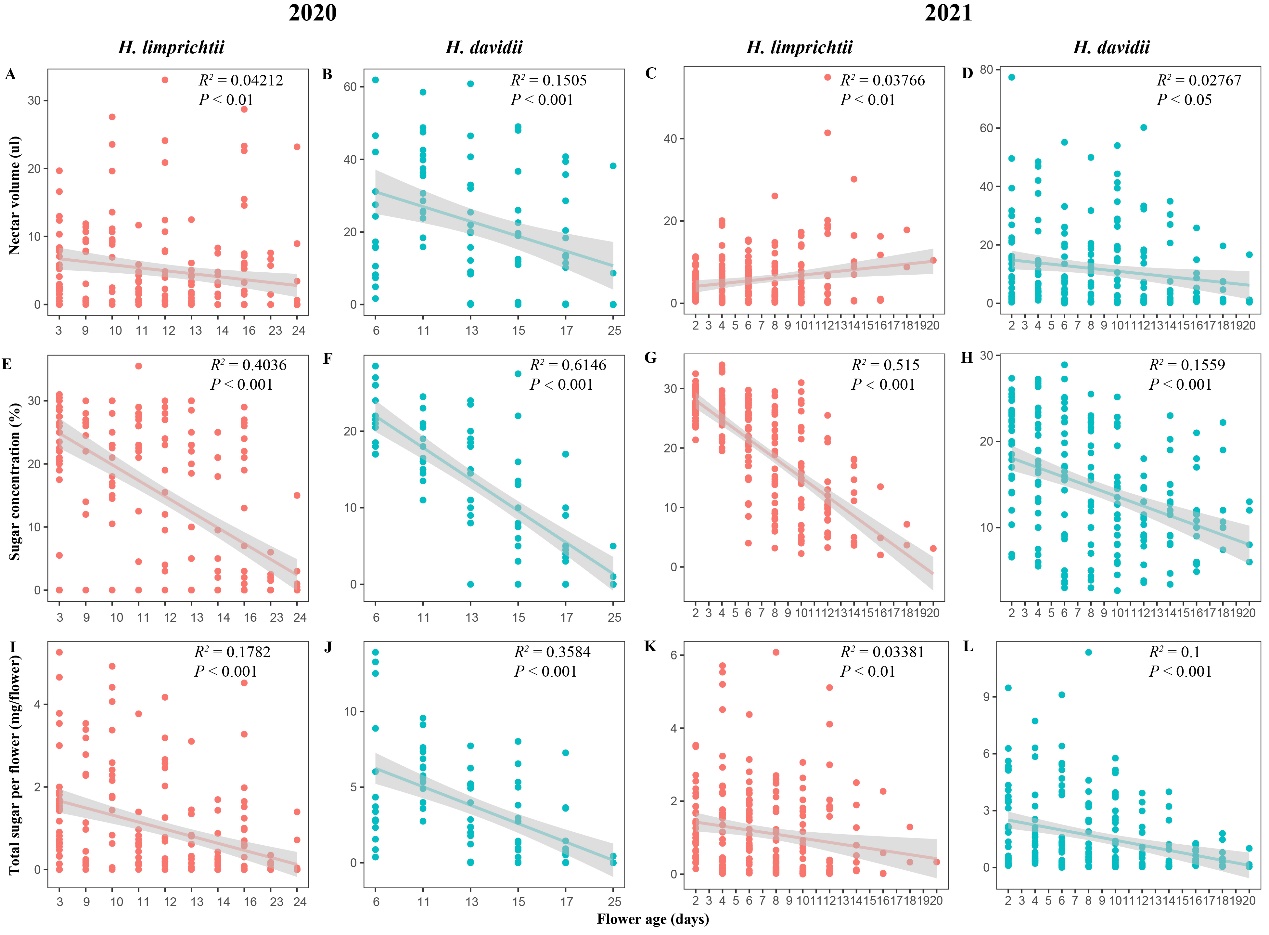


**Fig. S1** Relationship between nectar traits and flower age in *Habenaria limprichtii* and *H. davidii*. **A-D** Relationship of the mean nectar volume to flower age of two *Habenaria* species in 2020 and 2021. Relationship between nectar volume and flower age in *H. limprichtii* **(A)** and *H. davidii* **(B)** in 2020; Relationship between nectar volume and flower age in *H. limprichtii* **(C)** and *H. davidii* **(D)** in 2021; **E-H** Relationship of the mean sugar concentration to flower age of two *Habenaria* species in 2020 and 2021. Relationship between sugar concentration and flower age in *H. limprichtii* **(E)** and *H. davidii* **(F)** in 2020; Relationship between sugar concentration and flower age in *H. limprichtii* **(G)** and *H. davidii* **(H)** in 2021; **I-L** Relationship between the total sugar per flower and flower age of two *Habenaria* species in 2020 and 2021. Relationship between the total sugar per flower and flower age in *H. limprichtii* **(I)** and *H. davidii* **(J)** in 2020; Relationship between the total sugar per flower and flower age in *H. limprichtii* **(K)** and *H. davidii* **(L)** in 2021.

**
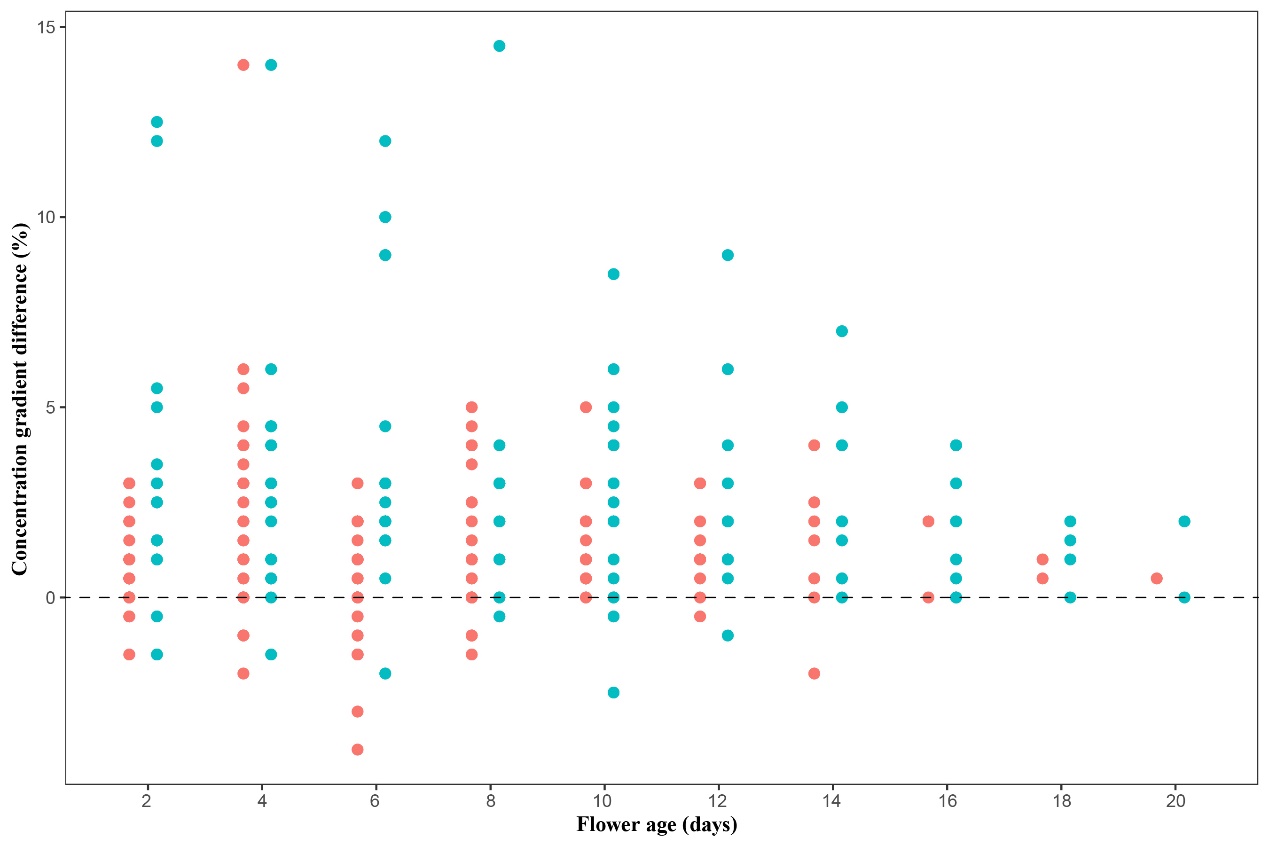
**

**Fig. S2** The variation in concentration gradient difference with flower age in two *Habenaria* species. Red circles represent the difference between the bottom and the top of the nectar column of *H. limprichtii*, and blue circles represent that of *H. davidii*.
